# Enhancing healthcare smartwatch adoption among older adults: perceptual affordance-based design recommendations for video demonstrations

**DOI:** 10.1093/geroni/igag047

**Published:** 2026-05-23

**Authors:** Hyo Jun Yang, Yu-Won Youn, Garyoung Kim, Ji-hyun Lee

**Affiliations:** Graduate School of Culture Technology, Korea Advanced Institute of Science and Technology, Daejeon, South Korea; Graduate School of Culture Technology, Korea Advanced Institute of Science and Technology, Daejeon, South Korea; Graduate School of Culture Technology, Korea Advanced Institute of Science and Technology, Daejeon, South Korea; Graduate School of Culture Technology, Korea Advanced Institute of Science and Technology, Daejeon, South Korea

**Keywords:** Technology acceptance, Perceptual affordances, UTAUT, Wearable technology

## Abstract

**Background and Objectives:**

Visual demonstrations play an important role in supporting older adults’ adoption of wearable healthcare technologies, but how they shape acceptance remains underexplored. This study aimed to identify the most suitable demonstration format for further investigation and, using the Unified Theory of Acceptance and Use of Technology (UTAUT) framework, examine how its features supported adoption.

**Research Design and Methods:**

Using an explanatory sequential mixed-methods design, Phase 1 involved a repeated-measures crossover survey (*N *= 62) comparing written, PowerPoint, video, and live demonstrations of smartwatch functions to guide the qualitative phase. Phase 2 involved semi-structured interviews (*N *= 16) to explore how video demonstrations, the highest-scoring format, activated 4 perceptual affordances—visibility, recordability, verifiability, and comparability. Transcripts were coded by UTAUT construct and affordance, followed by thematic analysis.

**Results:**

Phase 1 results showed that all enhanced demonstrations scored higher than the written baseline, with video achieving the highest descriptive means across UTAUT constructs. Phase 2 thematic analysis revealed three patterns: “Seeing Is Believing” described how clarity and visual verification fostered trust; “Finding Myself in the Demonstration” emphasized age-appropriate representation and contextual relevance; and “Perceived Family Approval in Technology Adoption” highlighted family validation and support as central to acceptance.

**Discussion and Implications:**

Contextual video segments were most effective for performance expectancy and social influence, whereas instructional close-ups often heightened perceived complexity, shaping effort expectancy and facilitating conditions. These findings informed 5 design recommendations: balance close-ups with contextual views, provide clear visual feedback, feature age-appropriate demonstrators, embed daily-life scenarios, and include family interactions to strengthen acceptance.

Innovation and Translational Significance: By linking perceptual affordances of video demonstrations, including visibility, recordability, verifiability, and comparability, to the constructs of the UTAUT, this study provides actionable strategies for practice. Specific design features, such as featuring older adult demonstrators, embedding demonstrations in daily-life scenarios, and balancing close-ups with contextual views, map directly onto key determinants of technology acceptance. These insights offer clear guidance for developers, clinicians, and educators to create demonstrations that reduce anxiety, build trust, and support older adults’ adoption of healthcare technologies.

## Background and objectives

Technology can help older adults with their health in many ways, such as monitoring health conditions, preventing the development of chronic conditions, and reducing caregiver burden (Czaja, 2017; Wang et al., 2016). Specifically, wearable devices are beneficial because they allow health-conscious consumers to track sleep patterns, heart rates, stress levels, oxygen uptake, and biochemical processes (Yildirim & Ali-Eldin, 2019). For older adults in particular, smartwatches can help with health monitoring by detecting health deterioration in a personalized way (Chung et al., 2022). Research has shown that wearable devices with fitness trackers and accelerometers increased physical activity among older adults (Cooper et al., 2018). Additionally, wearable devices that can make emergency calls are viewed as helpful for older adults and have high perceived benefits, as such technologies help relieve the burden of formal and informal caregivers and relatives (Wiegel et al., 2024). However, there is a significant gap between the need for assistive technologies and the actual adoption among older adults (Soar et al., 2020). Specifically, acceptance of wearable devices remains low among older adults (Talukder et al., 2020), largely due to age-related characteristics (e.g., hearing loss, limited dexterity, and low vision) and lower levels of technology self-efficacy (Abouzahra & Ghasemaghaei, 2020).

The Unified Theory of Acceptance and Use of Technology (UTAUT) was developed by Venkatesh et al. (2003) and has since become one of the most widely applied models for explaining technology acceptance. It is frequently used in the context of healthcare technologies as well (Rouidi et al., 2022). UTAUT explains behavioral intention (BI), the strongest predictor of actual technology use, through four key constructs. Performance expectancy (PE) refers to the degree to which an individual believes the technology will improve outcomes, while effort expectancy (EE) captures the perceived ease of using the technology. Social influence (SI) reflects the extent to which individuals perceive important others expect them to use the technology. Finally, facilitating conditions (FC) describe the degree to which individuals believe that supportive infrastructure or resources exist to enable use. In the original UTAUT model, FC was theorized to influence usage behavior directly rather than behavioral intention. However, later extensions, such as the senior technology acceptance model, demonstrated that for older adults, FC also influences attitudes toward using and technology acceptance (Chen & Chan, 2014; Chen & Lou, 2020).

Educational and digital literacy interventions have been shown to shape older adults’ technology acceptance beliefs by strengthening key UTAUT determinants (Fleischer et al., 2025; Yang et al., 2022). Within this broader instructional landscape, demonstrations, defined here as instructional techniques that illustrate how technologies function and in what contexts they may be used, can be understood as a lightweight educational strategy for influencing early acceptance beliefs by making system operations visible prior to hands-on use. Videos that explain the technology effectively gained older adults’ attention (Hansen et al., 2020; Smith et al., 2019) and have been used to improve intention to use health technology for older adults (Tam et al., 2017). In addition, live, hands-on demonstrations enhanced confidence and engagement with new technologies, underscoring the value of experiential exposure (Evans et al., 2018). Such strategies, by employing both auditory and visual modalities, can optimize cognitive channel allocation and improve learning efficiency (Mayer, 2017), particularly benefiting older adults experiencing age-related declines in working memory, attentional control, and digital literacy (Brockmole & Logie, 2013). These approaches align with digital inclusion frameworks emphasizing accessible, experience-based strategies for bridging the technology adoption gap among older adults (Czaja et al., 2019), providing tailored instruction that ensures more equitable access to healthcare innovations (Cui et al., 2025; Hepburn et al., 2025).

Building on these findings, a recent meta-analysis of 41 UTAUT studies showed that visual demonstrations, including PowerPoint, video, and live formats, significantly improved behavioral intention toward healthcare technologies among older adults (Yang et al., 2025). However, the three formats were analyzed in aggregate, limiting insight into how different demonstration approaches may differentially influence acceptance. Moreover, prior work has rarely examined how specific design features within demonstrations shape key UTAUT constructs. Addressing this gap is important for enabling designers, researchers, and practitioners to develop demonstrations that more precisely support older adults’ technology acceptance.

Therefore, this study had three objectives:

1. To compare different types of visual demonstrations (PowerPoint, video, and live) in order to identify a format suitable for deeper qualitative investigation.
2. To examine which specific elements within the selected format shaped older adults’ perceptions across the UTAUT constructs.
3. To translate these insights into theoretically grounded and practically actionable design recommendations for creating effective visual demonstrations.

## Research design and methods

### Overview of research design

**Figure 1:**
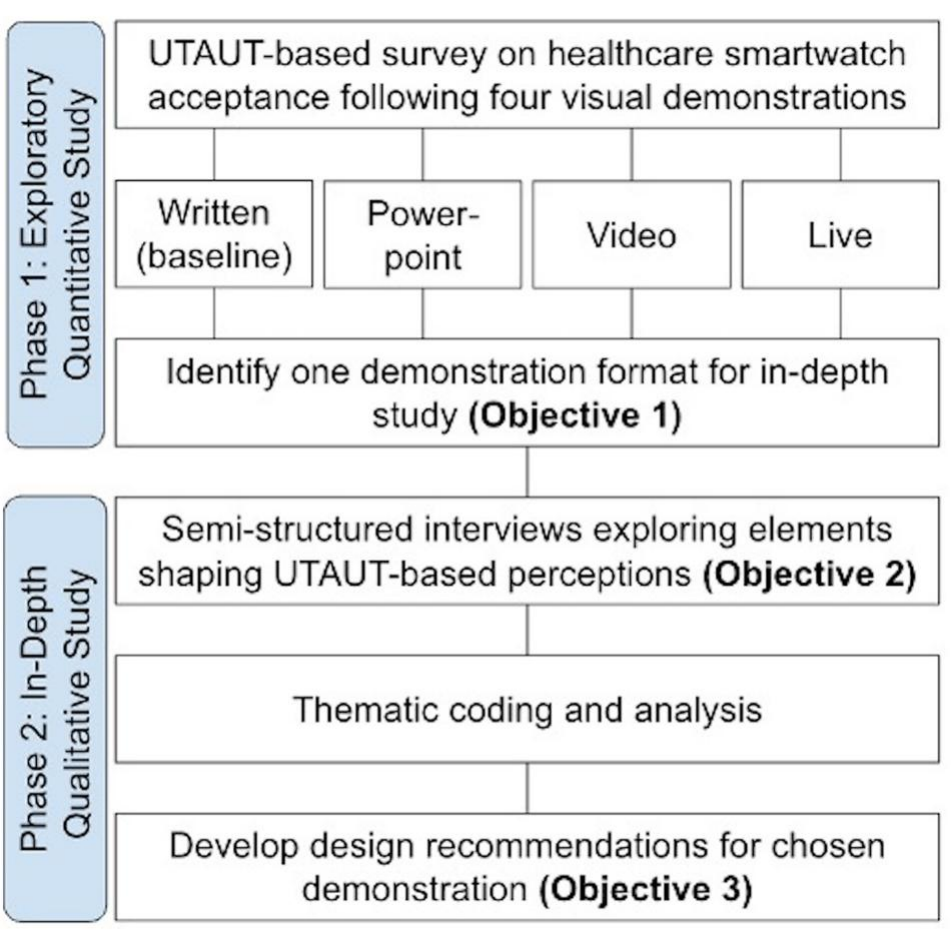
Research design overview aligned with study objectives. Note. UTAUT = Unified Theory of Acceptance and Use of Technology.

This study employed an explanatory sequential mixed-methods design, in which an initial quantitative phase provided preliminary insights that guided the focus of a subsequent qualitative phase (Toyon, 2021). The quantitative phase addressed Objective 1 by serving as an exploratory screening step to compare three visual demonstration formats, PowerPoint, video, and live, and to identify one format for deeper qualitative investigation. Statistical analyses (repeated-measures ANOVA with post-hoc tests) were conducted, but results were interpreted descriptively to highlight patterns rather than to establish confirmatory generalizations as the primary purpose of this phase was to inform the design and focus of the qualitative phase. Building on these results, the qualitative phase addressed Objectives 2 and 3 by examining participants’ perceptions of the selected format in greater depth, focusing on how specific features shaped the UTAUT constructs (PE, EE, SI, and FC), which are central to older adults’ intention to adopt technology, and by translating these insights into design recommendations. The research overview and objectives are illustrated in Figure 1. This study received approval from the Korea Advanced Institute of Science and Technology Institutional Review Board under two protocols: KH2024-254 (quantitative survey) and KAISTIRB-2025-71 (qualitative interviews).

### Phase 1: Quantitative methods

#### Study design and demonstration conditions

Phase 1 used a repeated-measures crossover survey design to compare four demonstration conditions: baseline written description, PowerPoint, video, and live presentation, each presenting identical smartwatch functions (heart rate monitoring, exercise tracking, and emergency SOS). The three formats were selected as they were shown to have a significant effect on UTAUT-based acceptance of technology (Yang et al., 2025). The PowerPoint condition presented static slides accompanied by narration from the interviewer. The video condition showed a pre-recorded walkthrough of the smartwatch with guided narration and subtitles. The live condition consisted of a real-time demonstration delivered by the interviewer using an actual smartwatch. All demonstrations were standardized in duration (2.5–3 min) and script length (830–850 Korean characters). The baseline consisted of a brief written information sheet with two sentences describing a healthcare smartwatch, approximating the minimal product descriptions older adults often encounter in brochures or pamphlets. While intentionally minimal to represent common real-world informational exposure, this condition may have accentuated contrasts with the enhanced demonstration formats. The order of conditions was counterbalanced across participants, and after each, participants completed the UTAUT-based acceptance survey.

#### Survey instrument and analysis plan

The survey consisted of two parts. First, a demographic questionnaire collected information on age, gender, education, living arrangement, employment status, income sources, and prior smartwatch experience. Second, technology acceptance was assessed using 18 items adapted from validated UTAUT instruments (Venkatesh et al., 2003), covering PE, EE, SI, FC, and BI. All items were rated on a 7-point Likert scale and adapted for accessibility with large fonts and high-contrast formatting. The questionnaire was administered four times: once at baseline after the written condition and again following each of the PowerPoint, video, and live demonstrations. See Supplementary Table 1 for the full questionnaire in English.

Although Phase 1 was exploratory in nature, standard statistical procedures commonly applied in UTAUT research were conducted to ensure rigor and comparability with prior studies. Reliability of the UTAUT questionnaire was assessed using Cronbach’s alpha, and construct validity was examined through confirmatory factor analysis, including checks for convergent validity (average variance extracted > 0.50) and composite reliability. Differences between demonstration formats were analyzed using repeated-measures ANOVA with Bonferroni-corrected pairwise comparisons. These tests were used as descriptive tools to identify general patterns across conditions rather than to establish definitive inferential claims, with the primary purpose of guiding the subsequent qualitative phase.

#### Participants and recruitment

Participants were recruited through convenience sampling from a large church in South Korea, which maintains an active senior ministry program. This setting was chosen due to the availability of a sizable older adult population, the familiarity and accessibility of the environment, and the presence of appropriate on-site spaces for repeated participation. Eligibility criteria required participants to be aged 65 or older with no prior experience using healthcare smartwatches, as the study focused on initial acceptance among new users. Screening was based on self-reported age and smartwatch experience. In addition, prior to participation, the researcher confirmed that participants could read the questionnaire text, hear verbal instructions, and follow the demonstration materials. Participants who showed difficulty understanding instructions or completing the survey due to visual, auditory, or cognitive limitations were allowed to participate but excluded from the analysis. All participants provided written informed consent prior to participation and received 20,000 KRW as compensation for their time.

### Phase 2: Qualitative methods

#### Design rationale and video demonstration design

Phase 2 employed a video demonstration, identified in Phase 1 as the most suitable format for further investigation, to examine how specific visual features, scenarios, and presentation styles shaped participants’ perceptions across the UTAUT constructs during semi-structured interviews. While detailed statistical comparisons of demonstration formats are presented in the *Results* section, the methods specific to Phase 2 are described here to maintain continuity and ensure readability. The video, identical to the one used in Phase 1, introduced three smartwatch functions (heart rate monitoring, exercise tracking, and emergency SOS), each presented through two complementary segments (an instructional segment and a contextual segment), resulting in six segments total. Instructional segments provided close-up, step-by-step demonstrations emphasizing procedural clarity, while contextual segments depicted the same functions within familiar routines such as morning walks, television viewing, and gardening. This dual-segment structure draws on multimedia learning evidence that clearly structured procedural instruction reduces cognitive overload and improves retention (Mayer & Chandler, 2001; Renkl, 2021), and on situated learning research showing that embedding skills in realistic contexts supports interpretation and transfer (Ackermans et al., 2019), both of which are particularly relevant for older adults who prefer visually supported, everyday-grounded instruction (Kim et al., 2023).

**Figure 2:**
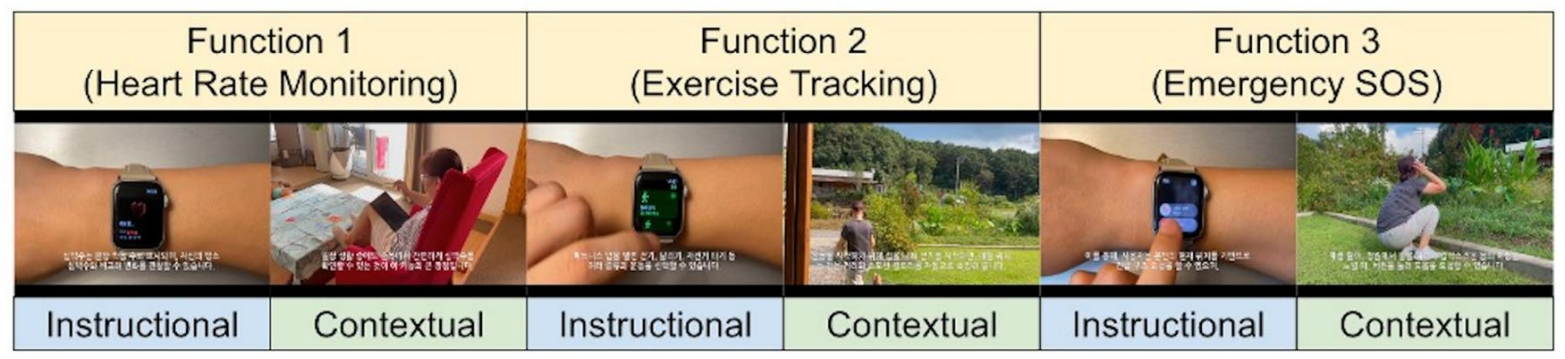
Video demonstration structure.

The design of the video was informed by prior research on creating older-adult–friendly instructional materials, which emphasizes appropriate pacing of visuals and speech, high-quality audio, and accessibility features such as high-contrast visuals, clear narration, and enlarged subtitles (Kim et al., 2023). It was then refined through a single pilot session with three older adults (aged 68, 69, and 77). Their feedback confirmed adequate hearing and vision accessibility and guided minor refinements to ensure that basic usability aspects were appropriate. A schematic overview of the segment structure is shown in Figure 2.

#### Interview protocol

Semi-structured interviews were conducted to probe participants’ thoughts on the video segment by segment. Each interview began with participants watching the entire video without interruption, after which they re-watched one segment at a time and responded to four guiding questions aligned with the UTAUT constructs: “What about this segment helped you think the smartwatch would be useful for maintaining or improving your health?” (PE); “What about this segment made you think you could use the smartwatch easily?” (EE); “What about this segment made you think people important to you, such as family or friends, would want you to use the smartwatch?” (SI); and “What about this segment made you think you would have the resources, knowledge, or support needed to use the smartwatch?” (FC). Follow-up questions encouraged participants to elaborate on particular scenes, visual details, or presentation styles. Behavioral Intention was not directly probed, as the qualitative phase focused on the antecedent constructs theorized to influence it (Venkatesh et al., 2003). Participants could provide multiple responses or indicate no response if a segment did not contribute to a given construct, ensuring all six segments were examined across all four constructs. Each interview lasted 25–45 min, was conducted in person, and audio-recorded with consent. Transcripts were produced verbatim and verified for accuracy by three researchers.

#### Framework of perceptual affordance

To organize the large volume of responses within an established theoretical framework, they were coded using Eriksson and Eriksson’s (2019) model of perceptual affordances. This model was selected because it was specifically developed to analyze how instructional videos communicate action and meaning through distinct perceptual features, making it particularly well-suited to examining how specific video elements shaped participants’ interpretations of smartwatch functions. In this framework, perceptual affordances are not general cognitive mechanisms but specific ways that live-action instructional videos communicate action: Visibility (making details discernible), Recordability (preserving time-based sequences), Verifiability (supporting confirmation of authenticity), and Comparability (enabling viewers to map demonstrations to their own potential actions). UTAUT conceptualizes acceptance as driven by cognitive evaluations of usefulness, ease, and social support, and such evaluations can be shaped by how users interpret information about a system. In video-based demonstrations, these interpretations are often guided by perceptual affordances that make certain aspects of action more visible, verifiable, or relatable. Thus, perceptual mechanisms may influence acceptance constructs by shaping how older adults appraise the functionality, usability, and social meaning of the technology prior to hands-on experience.

Coding captured both the type of affordance referenced and the segment type (instructional or contextual) in which it appeared, allowing responses to be categorized according to the specific video features participants identified as shaping their perceptions. For example, a participant noting that close-up shots made the heart rate function appear useful would be coded as a Visibility affordance under the instructional segment for PE. Each coded excerpt represented a distinct reference to a feature of the video demonstration, and participants could contribute multiple excerpts if they discussed different segments or constructs, meaning counts represent total references rather than unique participants. The frequency of coded references was counted for each combination of construct, affordance, and segment type, with higher counts interpreted as indicating greater salience and stronger participant associations with particular UTAUT constructs (Namey et al., 2008).

After transcripts were cleaned and segmented, three graduate-level researchers trained in qualitative methods and Eriksson and Eriksson’s framework independently coded responses. Analytic rigor was maintained through a negotiated consensus coding approach, where three researchers independently coded transcripts and resolved discrepancies in iterative meetings, with a senior researcher providing final determination for any unresolved ambiguities (O’Connor & Joffe, 2020). The team then conducted thematic analysis, supported by affinity diagramming: coded excerpts were printed, clustered, and reorganized in a collaborative workshop until stable themes emerged. These themes illustrated how perceptual affordances linked specific video features to older adults’ smartwatch acceptance and directly informed the design recommendations.

#### Participants and recruitment

Participants for Phase 2 were recruited separately from those in the quantitative phase through convenience sampling at senior clubs across South Korea, with different sites used to prevent overlap with Phase 1 participants. Eligibility criteria mirrored those of Phase 1, requiring participants to be aged 65 or older and to have no prior experience with smartwatches. All participants provided written informed consent and received 20,000 KRW as compensation for their time.

## Results

### Phase 1: Quantitative phase findings

#### Survey participant demographics

Of the 91 older adults who initially participated, 29 were excluded (20 due to prior smartwatch experience and 9 due to impairments or comprehension difficulties), yielding a final sample of 62. This sample size is consistent with prior studies employing repeated-measures designs to examine technology perceptions among older adults (e.g., Classen et al., 2021). Participants had a mean age of 75.3 years (*SD* = 5.7, range = 65–90) and were predominantly female (72.6%). Most lived with family (79%), while 21% lived alone. Educational attainment varied, with 9.7% having completed primary education, 37.1% secondary education, and 53.2% post-secondary education. The majority were retired (67.7%), while others engaged in part-time (21%) or full-time (11.3%) employment. Pensions were the primary source of income, supplemented by assets (25.8%), family support (21%), and wages (14.5%). See Supplementary Table 2 for full survey participant demographics.

#### Reliability and validity analysis

Confirmatory factor analysis supported the psychometric soundness of the UTAUT instrument across all demonstration conditions. Internal consistency reliability was confirmed through Cronbach’s alpha coefficients, with all constructs exceeding the recommended threshold of 0.70 (Nunnally & Bernstein, 1994). Composite reliability values for all constructs were above 0.80, and average variance extracted values surpassed the recommended cutoff of 0.50, indicating strong convergent validity. See Supplementary Tables 3–6 for full reliability and validity analysis results.

#### Comparison of demonstration methods

Repeated-measures ANOVA indicated that demonstration format significantly influenced perceptions across all UTAUT constructs (all *F* > 13.9, *p* < .001; see Supplementary Table 7). Effect sizes were consistently in the medium-to-large range (partial η^2^ = .186–.218), underscoring the substantive impact of demonstration format on technology acceptance. Bonferroni-corrected post-hoc tests showed that all enhanced formats (PowerPoint, video, live) scored significantly higher than the baseline written condition across every construct (all *p* < .01). Comparisons among the enhanced formats were less consistent, though video demonstrations yielded the highest descriptive means for performance expectancy (*M* = 6.25), social influence (*M* = 6.22), effort expectancy (*M* = 5.77), and facilitating conditions (*M* = 4.79; see Supplementary Table 8). However, post-hoc comparisons did not indicate statistically significant differences among the enhanced formats. Therefore, video demonstrations were selected as a practical focus for the qualitative phase based on their consistently higher descriptive patterns rather than statistical superiority. Full post-hoc details are provided in Supplementary Table 9. Taken together, these findings supported the decision to examine video demonstrations more closely in order to address Objective 1.

### Phase 2: Qualitative findings

#### Interview participants demographics

Table 1 summarizes the demographic profile of the 16 participants who took part in the qualitative phase. Originally, 17 participants were recruited, but one was excluded due to vision issues that would have impacted video comprehension. Sixteen participants were deemed sufficient as repetition across responses indicated that data saturation had been reached, consistent with prior methodological discussions noting that as few as 16 interviews can be adequate for relatively homogeneous groups when no new themes emerge (Aguboshim, 2021). All participants had adequate vision and hearing capabilities and no prior experience with healthcare smartwatches. Compared to the survey sample, interview participants had a slightly higher mean age of 76.8 years (*SD* = 6.2) and a larger proportion of females (81.3%), with broadly similar distributions across living arrangements, education, occupation, and income sources (see Table 1).

**Table 1:** Demographic characteristics of interview participants (*N *= 16).

Characteristic	Value
**Age, in years**	
Mean (*SD*)	76.8 (6.2)
Range (min-max)	68-88
**Gender, *n* (%)**	
Male	3 (18.8)
Female	13 (81.3)
**Living arrangement, *n* (%)**	
Living with family	12 (75.0)
Living alone	4 (25.0)
**Education level, *n* (%)**	
Post-secondary education	7 (43.8)
Secondary education (middle/high school)	7 (43.8)
Primary education	2 (12.5)
**Current occupation, *n* (%)**	
Part-time work	4 (25.0)
Retired/not working	10 (62.5)
Full-time work	2 (12.5)
**Primary source of income, *n* (%)^a^**	
Pension/retirement benefits	11 (68.8)
Asset income^b^	5 (31.3)
Support from spouse/children/grandchildren/relatives	4 (25.0)
Wages^c^	3 (18.8)

Note. Percentages are based on the total number of participants unless otherwise indicated.

a Multiple responses possible.

b Includes savings, investments, or property income.

c Includes temporary or casual wages.

#### Overview of coded responses

A total of 96 coded responses were identified across all interviews, with 61 linked to contextual segments and 35 to instructional segments. These were distributed across the four UTAUT constructs and four perceptual affordances. By construct, EE accounted for the highest number of responses (37), followed by PE (28), FC (17), and SI (14). Instructional segments contributed 21 of the 37 EE responses, while contextual segments accounted for 22 of the 28 PE responses and 12 of the 14 SI responses. In terms of affordance type, comparability was the most frequently coded (37), followed by visibility (33), recordability (15), and verifiability (11). Comparability and visibility were cited in both instructional and contextual segments, with a higher number of comparability responses in contextual segments (27 out of 37). Table 2 summarizes the full distribution of coded responses by UTAUT construct, segment type, and perceptual affordance. These coding patterns addressed Objective 2 by showing how features of the video demonstration were linked to UTAUT constructs through different perceptual affordances.

**Table 2:** Frequency of responses by perceptual affordance and UTAUT construct.

UTAUT construct	Segment type	Visibility	Recordability	Verifiability	Comparability
**Performance expectancy**	Instructional	2	3	1	0
	Contextual	5	3	4	10
**Effort expectancy**	Instructional	8	4	2	7
	Contextual	5	2	1	8
**Social influence**	Instructional	1	0	0	1
	Contextual	4	1	1	6
**Facilitating conditions**	Instructional	3	1	0	2
	Contextual	5	1	2	3

Note. UTAUT = Unified Theory of Acceptance and Use of Technology. Values are frequencies of coded responses.

#### Performance expectancy

A total of 28 responses were coded for PE, with 6 from instructional segments and 22 from contextual segments. Most were associated with comparability and Visibility affordances. When asked about PE, participants most often described how the demonstrations showed the smartwatch contributing to health routines, such as step tracking or other daily activities. Instructional segments highlighted individual functions, whereas contextual segments illustrated broader benefits by situating the device within everyday scenarios.

“The subtitles told me that I can check my pulse whenever I want.” (Case 1, Female, 75) [Visibility affordance – Instructional segment]

“I walk every morning just like that woman. I could use it the same way to track my steps.” (Case 3, Female, 73) [Comparability affordance – Contextual segment]

“Watching her step count go up while she walked - that would motivate me to take more steps.” (Case 9, Female, 76) [Visibility affordance – Contextual segment]

#### Effort expectancy

A total of 37 responses were coded for EE, with 21 linked to instructional segments and 16 to contextual segments. Most of these responses were associated with Visibility and Comparability affordances. When asked about EE, participants frequently described how the clarity of interface elements, button sizes, or interaction steps shaped their perceptions of ease or difficulty. Instructional segments often revealed usability barriers in detail, while contextual segments illustrated how these interactions might unfold in everyday situations.

“Those buttons for the heart rate app looked tiny in the close-up. Old people cannot press things like that.” (Case 7, Female, 72) [Visibility affordance – Instructional segment]

“Their hands were so steady using the SOS button. Old people’s hands shake.” (Case 14, Male, 88) [Comparability affordance – Instructional segment]

“The walking results showed too many different numbers at once. Why can’t it just focus on one thing at a time?” (Case 16, Female, 71) [Visibility affordance – Contextual segment]

#### Social influence

A total of 14 responses related to SI were coded, with 2 linked to instructional segments and 12 to contextual segments. Comparability was the most frequently associated affordance. These responses often involved participants reflecting on how others might respond to their smartwatch use. Instructional segments prompted occasional personal associations, but contextual segments more effectively elicited reflections on family support and approval by embedding the device in social settings.

“Watching the heart monitor tutorial reminded me of my grandson. He tries to teach me about my phone and health functions as well. He might like this.” (Case 15, Female, 74) [Comparability affordance – Instructional segment]

“My children will be happy that I am taking their advice and take care of my health when we take a walk together.” (Case 6, Female, 78) [Comparability affordance – Contextual segment]

“I thought how relieved my daughter would be knowing she’d get alerted if I had a fall.” (Case 2, Female, 80) [Verifiability affordance – Contextual segment]

#### Facilitating conditions

A total of 17 responses related to FC were coded, with 6 for instructional segments and 11 for contextual segments. These responses were primarily associated with Visibility and Comparability affordances. Participants discussed whether they could use the device independently or would require assistance, often prompted by demonstrations of complex steps or small interface elements. Instructional segments highlighted potential operational challenges, while contextual segments encouraged participants to envision the types of support or resources that might be needed in practice.

“The way they showed the heart rate buttons, I need someone to remind me which one to press the first few times.” (Case 3, Female, 73) [Visibility affordance – Instructional segment]

“The small print on the heart rate screen is problematic. There needs to be an instruction manual to explain if there’s a way to enlarge the display text.” (Case 9, Female, 76) [Visibility affordance – Contextual segment]

“I had a problem setting up my TV and had to call their customer service. Is there something like that for the watch?” (Case 6, Female, 78) [Comparability affordance – Contextual segment]

### Qualitative findings: thematic analysis

The analysis yielded three major themes, each with two subthemes, illustrating how specific affordances shaped older adults’ perceptions across the UTAUT constructs. The themes are presented below with illustrative quotes.

#### Theme 1: “Seeing Is Believing” (33 responses)

This theme captures how visual elements in demonstrations influenced participants’ trust and understanding of the technology.

Subtheme 1.1: Clarity vs. complexity in visual presentations: Visibility Affordance for Effort Expectancy (21 responses).

“The close-up showed exactly where to tap for the exercise tracker.” (Case 3, Female, 73)

“Those buttons for the heart rate app looked tiny in the close-up. Old people cannot press things like that.” (Case 7, Female, 72)

Close-up visual demonstrations created a paradoxical effect for many participants. While they appreciated the clear visibility of interface details, this same clarity often highlighted the complexity and precision required, especially for those with physical limitations such as hand tremors or visual impairments. This subtheme reveals how the Visibility affordance can both enhance understanding and increase perceived difficulty simultaneously.

Subtheme 1.2: Visual verification building confidence: Verifiability affordance for performance expectancy (12 responses).

“When I actually saw the heart rate number changing on the screen, that’s when I thought this actually works.” (Case 6, Female, 78)

“Seeing those heart rate numbers actually change after she turns it on showed me it really works.” (Case 14, Male, 88)

Participants frequently mentioned how seeing immediate cause-and-effect relationships in demonstrations built their confidence in the technology’s functionality. This subtheme highlights how the Verifiability affordance operates through visual confirmation, particularly in contextual segments showing the technology being used in realistic situations.

#### Theme 2: “Finding Myself in the Demonstration” (26 responses)

This theme encompasses participants’ experiences of relating personally to what they observed in the demonstrations.

Subtheme 2.1: Age-appropriate representation matters: Comparability affordance for Effort Expectancy (13 responses).

“That lady was about my age but handled the heart monitor easily. It makes me think maybe I could too.” (Case 8, Female, 69)

“Their hands were so steady using the SOS button. Old people’s handshake.” (Case 14, Male, 88)

Participants were highly sensitive to the age and physical characteristics of demonstrators. When demonstrators resembled participants in age and abilities, this activated strong comparability affordances, enhancing both performance expectancy and effort expectancy. Conversely, younger demonstrators often created negative comparisons that reduced confidence.

Subtheme 2.2: Contextual relevance to daily life: Comparability affordance for performance expectancy (13 responses).

“I walk every morning just like that woman. I could use it the same way to track my steps.” (Case 3, Female, 73)

“That SOS feature is something I can use because I am scared of falling in the bathroom. Many older adults have that fear, you know?” (Case 13, Female, 79)

When demonstrations depicted scenarios that mirrored participants’ daily routines and environments, they more readily envisioned the technology’s benefits in their own lives. This subtheme illustrates how contextual relevance operates through comparability affordances to enhance performance expectancy by making benefits concrete rather than abstract.

Theme 3: “Perceived Family Approval in Technology Adoption” (18 responses)

This theme captures how participants’ perceptions of technology were significantly shaped by anticipated family reactions and social dynamics.

Subtheme 3.1: Family validation through contextual demonstrations: Comparability affordance for social influence (10 responses).

“I think this process of checking my exercise information after walking is something my family wants me to do.” (Case 1, Female, 75)

“My children will be happy that I am taking their advice and take care more of my health when we are walking together.” (Case 6, Female, 78)

Contextual segments depicting technology use in family settings prompted participants to consider how using the device might affect their relationships with family members. The first-person point-of-view camera angle in these segments helped participants imagine themselves within these family interactions, enhancing the sense of personal relevance. This subtheme reveals how demonstrations activated Social Influence primarily through comparability affordances that helped participants visualize family approval and concern in their own lives.

Subtheme 3.2: Anticipated family assistance as acceptance factor: comparability affordance for facilitating conditions (eight responses).

“I need someone to tell me to remember to turn the exercise function before I begin.” (Case 4, Female, 82)

“My son’s always helping me with technology, and I need him to show me the heart rate thing a few times.” (Case 7, Female, 72)

Participants frequently referenced potential family support when evaluating the technology, particularly after viewing scenes depicting intergenerational assistance. This consideration often transformed perceived barriers into manageable challenges, as participants envisioned family members providing the necessary support to overcome initial difficulties. The subtheme highlights how anticipated family assistance served as both a practical facilitating condition and an emotional reassurance that enhanced overall technology acceptance.

### Additional perceptual considerations (19 responses)

“When she was sitting checking her heart rate, the watch face looked too dark. I prefer brighter displays even if it uses more battery.” (Case 10, Male, 70)

“The walking results showed too many different numbers at once. Why can’t it just focus on one thing at a time?” (Case 16, Female, 71)

“I had a problem setting up my TV and had to call their customer service. Is there something like that for the watch?” (Case 6, Female, 78)

In addition to the three main themes, participants also expressed practical concerns and observations that didn’t fit neatly into thematic categories. These included technical preferences, interface and accessibility concerns, and questions about technical support.

## Discussion and implications

### Differential impact of video segments on technology acceptance

Differences emerged in how participants responded to contextual and instructional segments. Contextual segments proved especially effective for supporting PE, SI, and FC: participants envisioned themselves using the smartwatch in familiar routines, activating Comparability affordances that helped them map demonstrations onto their own lives, while Visibility and Verifiability reinforced trust by clearly showing device benefits in action. Embedding the technology in family and social contexts further encouraged reflections on approval from children or peers and on available assistance.

Instructional segments, by contrast, most often shaped EE. Close-up demonstrations made operational steps highly visible, but this same clarity sometimes magnified concerns about button size, shaky hands, or screen complexity. Contextual segments also raised effort-related concerns, though less intensely, when participants compared their own abilities with those of demonstrators. Overall, instructional demonstrations were valuable for teaching specific steps but, when presented alone, risked overwhelming viewers with precision and complexity.

These patterns align with prior research showing that older adults prefer learning content connected to daily life rather than abstract procedures (Hawley-Hague et al., 2014; Sharpe & Elwood, 2024), that perceptual and cognitive changes can make complex instructional formats more difficult to process (Charness & Boot, 2009), and that excessive detail may increase technology-related anxiety (Vaportzis et al., 2017). Taken together, the findings suggest that instructional segments are most effective when paired with contextual portrayals, which provide the concreteness and relevance needed to support technology acceptance among older adults.

### Design recommendations for video demonstrations

For Objective 3, five hypothesis-generating design recommendations (Table 3) were developed based on the thematic findings, extending existing usability guidelines by addressing perceptual factors that may shape older adults’ technology acceptance.

**Table 3:** Design recommendations of video demonstrations based on thematic analysis.

Affordance type	Recommendations (how)	Issue (what)	Rationale for recommendations (why)
**Visibility**	(V1) Use medium close-ups combined with wider contextual views	Close-ups highlight complexity and overwhelm some viewers	Extreme close-ups unintentionally increased perceived difficulty (Theme 1.1). Balancing close-ups with wider views reduces anxiety while preserving clarity.
**Verifiability**	(F1) Show immediate system feedback after user interaction	Older adults are unsure if the function works without proof	Visual feedback (e.g., heart rate number changing) confirmed functionality for participants (Theme 1.2). Builds trust in the device.
**Comparability**	(C1) Use age-appropriate demonstrators with visible age-related characteristics	Older adults struggle to relate to young or highly capable demonstrators	Participants reported reduced confidence when demonstrators seemed younger or more skilled (Theme 2.1). Matching demonstrator characteristics improves Comparability and increases perceived self-efficacy (PE, EE).
**Comparability**	(C2) Ground demonstrations in realistic, daily-life scenarios	Contextual scenarios that resonate with older adults’ daily experiences elicit the most positive perceptions	Participants better understood benefits when they could map scenarios to their routines (Theme 2.2). This strengthens Performance Expectancy through relatable use cases.
**Comparability**	(C3) Include family interaction and intergenerational support scenes	Family acceptance and support is a crucial factor in perception of technology	Social Influence was more salient in contextual videos depicting family dynamics (Themes 3.1, 3.2). These scenes promote motivation through perceived support and approval.

Note. EE = effort expectancy; PE = performance expectancy.

### Use medium close-ups with contextual balance (V1)

The analysis showed that extreme close-ups, while clarifying procedural details, often magnified usability concerns such as small button size, shaky hands, or crowded screens (Theme 1.1). This discouraged confidence by making interactions appear more difficult than intended. To reduce this effect, demonstrations may benefit from medium close-ups that preserve clarity while balancing them with wider contextual views. This approach situates the interaction in daily life without overemphasizing technical precision. Prior research supports this adjustment, indicating that excessive visual detail can increase technology-related anxiety and cognitive load among older adults (Charness & Boot, 2009; Vaportzis et al., 2017), an important consideration given that self-efficacy is a significant predictor of technology adoption (Berkowsky et al., 2018). Within the UTAUT framework, this recommendation strengthens EE by reducing perceived barriers to use.

### Provide clear visual feedback (F1)

Participants consistently expressed greater trust when demonstrations showed immediate outcomes, such as heart rate values changing in real time (Theme 1.2). This visible feedback reassured them that the smartwatch worked as intended and reduced doubts about its reliability. To build such confidence, video demonstrations may emphasize clear visual feedback that verifies functionality. This directly supports PE, as users perceive the technology as effective in achieving health goals. Prior work confirms that trust is a critical barrier to technology acceptance among older adults (Yusif et al., 2016) and that clear visual feedback can alleviate such concerns by enhancing transparency (Mujirishvili et al., 2023). Similarly, video instructions have been shown to be particularly effective when they clearly display both step sequences and outcomes, helping older adults verify that the technology functions as intended (Pryor & McLaughlin, 2018).

### Feature age-appropriate demonstrators (C1)

Participants frequently compared themselves to the demonstrators. When demonstrators were perceived as younger or physically more capable, participants expressed doubt about their own ability to use the smartwatch (Theme 2.1). By contrast, age-appropriate demonstrators fostered relatability and strengthened self-efficacy through Comparability affordances. Therefore, video demonstrations may benefit from featuring models who are older adults themselves and who realistically represent physical capabilities common in this population. This approach builds EE and reduces stereotype threat by allowing older adults to envision themselves succeeding with the technology. This finding resonates with prior research showing that learners often prefer instructors who share similar demographic characteristics, such as age, gender, or ethnicity (Brooks et al., 2018; Dee, 2005), and aligns with studies emphasizing these preferences in the context of older adults and video-based instruction (Kim et al., 2023; Ma et al., 2020).

### Ground demonstrations in daily-life scenarios (C2)

When demonstrations were embedded in authentic daily routines, participants more readily envisioned how the smartwatch could enhance their own health practices (Theme 2.2). These contextual portrayals activated PE by making benefits concrete rather than abstract, while also supporting facilitating conditions by prompting participants to consider available support structures. To maximize acceptance, video demonstrations could be grounded in scenarios that older adults can easily connect to their own lives. This finding is consistent with prior research demonstrating that the adoption and sustained use of technologies is closely linked to their perceived relevance to daily routines (Hawley-Hague et al., 2014; Kim et al., 2023), and further extends to preferences for instructional videos (Kim et al., 2023).

### Include family interaction scenes (C3)

Many participants evaluated the smartwatch in terms of how family members might approve of, encourage, or assist with its use (Themes 3.1 and 3.2). Contextual scenes depicting family validation or intergenerational support activated SI and FC, reassuring participants that they would not be alone in learning the device. Video demonstrations could therefore include family interaction scenes that portray both encouragement and assistance as part of normal routines. Prior research confirms that family plays a central role in older adults’ technology adoption, creating both value and confidence (Ahmad et al., 2022; Lai, 2020). However, these portrayals must avoid reinforcing dependency. Family should be depicted as partners in health rather than caregivers, thereby supporting autonomy while still acknowledging available support (Li et al., 2020). The prominence of family influence observed in this study may reflect the collectivist cultural context of South Korea, where family involvement often plays a central role in health-related decision making among older adults.

### Limitations

This study has several limitations that should be acknowledged. First, it examined a single technology, a health smartwatch, which may limit generalizability to other healthcare devices or platforms. Technologies such as glucose monitors, blood pressure cuffs, or telehealth systems differ in interaction demands and perceived benefits, and the present findings cannot be assumed to transfer directly. Second, participants were recruited through convenience sampling, including a church community for Phase 1 and several senior centers for Phase 2, all located in a relatively affluent area of South Korea. These groups may represent older adults who are more socially active and receptive to technology than the general population, and the voluntary nature of participation may have attracted individuals with higher baseline interest, potentially inflating acceptance scores. Third, the cultural context of South Korea, where collectivist values and strong family orientation are prominent, may have influenced participants’ responses. The emphasis on family validation and anticipated support observed in the findings may be less pronounced in more individualistic societies, limiting cross-cultural generalizability.

## Conclusion

This study examined how video demonstrations influence older adults’ acceptance of wearable healthcare technology. The exploratory quantitative phase showed that video demonstrations had the highest descriptive means across acceptance constructs, guiding their selection for qualitative analysis. The qualitative findings revealed how specific video features, such as close-ups, daily-life scenarios, and visible system feedback, shaped UTAUT constructs: PE and SI were strengthened by everyday relevance and family approval, while EE and FC were shaped by the clarity and complexity of operational steps. By aligning demonstration design with perceptual affordances, developers and educators can reduce barriers, highlight benefits, and promote the adoption of health technologies that support independence and ease caregiver burden. Future studies could experimentally manipulate specific design features, such as segment type, close-up framing, and demonstrator age, to establish causal relationships with older adults’ technology acceptance constructs.

## Supplementary Material

igag047_Supplementary_Data

## Data Availability

Because of identifiable information in the data used in this study, it will not be made available for other researchers. This study was not preregistered.
