# Supplementary material for: Enhancing healthcare smartwatch adoption among older adults: perceptual affordance-based design recommendations for video demonstrations
Source: Innov Aging. 2026 May 23;10(8):igag047. doi: 10.1093/geroni/igag047 (PMC13329077; doi:10.1093/geroni/igag047)
Supplement: igag047_Supplementary_Data [file igag047_supplementary_data.pdf]

***Innovation in Aging* Supplementary Material: Yang, Youn, Kim, & Lee. Enhancing Healthcare Smartwatch Adoption Among Older Adults: Perceptual Affordance-Based Design Recommendations for Video Demonstrations.**

Supplementary Table 1. Full Questionnaire Items for Survey

Construct	Item	Question
**Performance Expectancy (PE)**	PE1	I think a healthcare smartwatch would be useful in my daily life.
	PE2	Using a healthcare smartwatch would allow me to manage my health better.
	PE3	Using a healthcare smartwatch would make health management faster and easier.
	PE4	Using a healthcare smartwatch would improve the efficiency of my health management.
**Effort Expectancy (EE)**	EE1	I think it would be easy for me to learn how to use a healthcare smartwatch.
	EE2	The healthcare smartwatch has clear and understandable instructions.
	EE3	I think using a healthcare smartwatch would not be difficult.
	EE4	I think I could easily become skilled at using a healthcare smartwatch.
**Social Influence (SI)**	SI1	People who are important to me think that I should use a healthcare smartwatch.
	SI2	People around me will say that I should use a healthcare smartwatch.
	SI3	People I trust would want me to use a healthcare smartwatch.
**Facilitating Conditions (FC)**	FC1	I have the equipment or resources necessary to use a healthcare smartwatch.
	FC2	I have sufficient knowledge necessary to use a healthcare smartwatch.
	FC3	The healthcare smartwatch is compatible with other devices I use.
	FC4	If I encounter difficulties while using the healthcare smartwatch, I can get help.
**Behavioral Intention (BI)**	BI1	I intend to use a healthcare smartwatch in the future.
	BI2	I will always try to use a healthcare smartwatch in my daily life.
	BI3	I will use a healthcare smartwatch in the future.

Supplementary Table 2. Demographic Characteristics of Survey Participants (N=62)

Characteristic	Value
Participants, n	62
Age (years), mean (SD)	75.3 (5.7)
Age range (min-max)	65-90
**Gender**	
Male	17 (27.4%)
Female	45 (72.6%)
**Living arrangement**	
Living with family	49 (79%)
Living alone	13 (21%)
**Education level**	
Post-secondary education	33 (53.2%)
Secondary education (middle/high school)	23 (37.1%)
Primary education	6 (9.7%)
**Current occupation**	
Part-time work	13 (21%)
Retired/Not working	42 (67.7%)
Full-time work	7 (11.3%)
**Primary source of income**[a]	
Pension/Retirement benefits	39 (62.9%)
Asset income[b]	16 (25.8%)
Support from spouse/children/grandchildren/relatives	13 (21%)
Wages[c]	9 (14.5%)
Local government or community subsidies	1 (1.6%)

Note. SD = standard deviation. Percentages are based on the total number of participants unless otherwise indicated.

[a]Multiple responses possible.

[b]Includes savings, investments, or property income.

[c]Includes temporary or casual wages.

Supplementary Table 3. Psychometric properties of UTAUT constructs under baseline (written) demonstration condition

Construct	No. of items	Cronbach's α	Composite Reliability (CR)	Average Variance Extracted (AVE)
**Performance Expectancy**	4	.87	.88	.67
**Effort Expectancy**	4	.86	.85	.59
**Social Influence**	3	.81	.82	.61
**Facilitating Conditions**	4	.83	.85	.60
**Behavioral Intention**	3	.93	.93	.81

Supplementary Table 4. Psychometric properties of UTAUT constructs under PowerPoint demonstration condition

Construct	No. of items	Cronbach's α	Composite Reliability (CR)	Average Variance Extracted (AVE)
**Performance Expectancy**	4	.94	.94	.81
**Effort Expectancy**	4	.95	.96	.84
**Social Influence**	3	.92	.92	.80
**Facilitating Conditions**	4	.88	.88	.65
**Behavioral Intention**	3	.90	.90	.76

Supplementary Table 5. Psychometric properties of UTAUT constructs under live demonstration condition

Construct	No. of items	Cronbach's α	Composite Reliability (CR)	Average Variance Extracted (AVE)
Performance Expectancy	4	.91	.91	.72
Effort Expectancy	4	.95	.95	.85
Social Influence	3	.88	.89	.74
Facilitating Conditions	4	.86	.87	.64
Behavioral Intention	3	.91	.91	.77

Supplementary Table 6. Psychometric properties of UTAUT constructs under video demonstration condition

Construct	No. of items	Cronbach's α	Composite Reliability (CR)	Average Variance Extracted (AVE)
Performance Expectancy	4	.91	.91	.72
Effort Expectancy	4	.96	.96	.86
Social Influence	3	.88	.89	.74
Facilitating Conditions	4	.88	.89	.66
Behavioral Intention	3	.95	.95	.87

Supplementary Table 7. Repeated-measures ANOVA results for UTAUT constructs

Construct	*F*(3, 183)	*p*	Partial η²
PE	16.97	< .001	.22
EE	13.93	< .001	.19
SI	15.77	< .001	.21
FC	14.19	< .001	.19
BI	14.26	< .001	.19

Note. PE = Performance Expectancy; EE = Effort Expectancy; SI = Social Influence; FC = Facilitating Conditions; BI = Behavioral Intention. Partial η² values are rounded to two decimals.

Supplementary Table 8. Descriptive statistics (M ± SD) of UTAUT constructs by demonstration format

Construct	BL (Written)	PP (PowerPoint)	VD (Video)	LD (Live)
PE	5.57 ± 1.19	6.18 ± 1.14	6.25 ± 1.04	6.10 ± 1.15
EE	4.96 ± 1.24	5.64 ± 1.21	5.77 ± 1.17	5.64 ± 1.36
SI	5.22 ± 1.30	5.98 ± 1.19	6.22 ± 0.90	6.01 ± 1.17
FC	3.82 ± 1.63	4.69 ± 1.68	4.79 ± 1.64	4.43 ± 1.77
BI	5.06 ± 1.65	5.88 ± 1.39	5.92 ± 1.45	5.88 ± 1.53

Note. BL = Baseline (written); PP = PowerPoint; VD = Video; LD = Live.Values are presented as M ± SD.

Supplementary Table 9. Bonferroni-corrected pairwise comparisons of demonstration formats

Comparison	PE (ΔM, *p*)	EE (ΔM, *p*)	SI (ΔM, *p*)	FC (ΔM, *p*)	BI (ΔM, *p*)
BL – PP	-0.61, < .001	-0.68, < .001	-0.76, < .001	-0.87, < .001	-0.81, < .001
BL – VD	-0.69, < .001	-0.81, < .001	-1.01, < .001	-0.97, < .001	-0.86, < .001
BL – LD	-0.54, < .001	-0.68, < .001	-0.80, < .001	-0.60, < .01	-0.81, < .001
PP – VD	-0.08, = 1.000	-0.12, = 1.000	-0.24, = .750	-0.10, = 1.000	-0.05, = 1.000
PP – LD	0.07, = 1.000	0.00, = 1.000	-0.03, = 1.000	0.27, = .631	0.00, = 1.000
VD – LD	0.15, = .985	0.13, = 1.000	0.21, = 1.000	0.36, = .165	0.05, = 1.000

Note. PE = Performance Expectancy; EE = Effort Expectancy; SI = Social Influence; FC = Facilitating Conditions; BI = Behavioral Intention. Values represent mean differences (ΔM) between demonstration formats with Bonferroni-adjusted p-values.
